# The elusive link between high sensitivity C-reactive protein and carotid subclinical atherosclerosis in coronary artery bypass grafting candidates: A cross-sectional study

**DOI:** 10.1186/1476-7120-6-23

**Published:** 2008-05-30

**Authors:** Parvin Shakouri, Nariman Nezami, Mohammad Kazem Tarzamni, Reza Javad Rashid

**Affiliations:** 1 Department of Radiology, Tabriz University of Medical Sciences, Tabriz, Iran; 2 Young Researchers Club, Tabriz Islamic Azad University, Tabriz, Iran; 3 Drug Applied Research Center, Tabriz University of Medical Sciences, Tabriz, Iran

## Abstract

**Background:**

Previous studies demonstrated a modest association between C-Reactive Protein (CRP), stenosis of carotid artery, and carotid Intima-Media Thickness (IMT) in general population. During present study, we aimed to evaluate the relationship between High Sensitivity C-Reactive Protein (hsCRP) and Common Carotid Intima-Media Thickness (CCIMT) in patients who candidate for Coronary Artery Bypass Grafting (CABG).

**Methods:**

The study subjects were enrolled from patients with coronary arteries disease referred from Shahid Madani Hospital (Tabriz, Iran), who have been candidate for elective CABG from January 2005 to August 2007. The common carotid arteries were evaluated with high-resolution B-mode ultrasonography using a 7.5- MHz linear-array transducer to determine the IMT and grade of stenosis. Serum hsCRP level was measured using commercially available enzyme linked immunosorbent assay kit.

**Results:**

Finally, information of 176 CABG candidates was analysed. The mean age of participants was 62.71 ± 9.45 years with 1.63 male to female ratio. The mean of CCIMT was 0.69 ± 0.54 mm. Although there was no significant correlation between serum hsCRP level and CCIMT in patients without carotid stenosis (p=0.113, r=0.186), participants with common carotid artery stenosis had higher levels of serum hsCRP than participants without stenosis (2.42+/-1.30 vs. 1.20+/-0.97 mg/dl; p=0.009).

**Conclusion:**

Study results showed that there was no correlation between serum hsCRP level and CCIMT in patients without carotid stenosis, but patients with common carotid artery stenosis had higher levels of serum hsCRP than patients without stenosis.

## Background

Assessment of carotid arteries stenosis and Intima-Media Thickness (IMT) by high resolution B-mode ultrasonography have been shown correlated with prevalence of cardiovascular disease, myocardial infarction, and stroke [1-3]. Some previous studies reported higher risk of myocardial infarction and stroke for patients who had higher levels of carotid arteries IMT [3,4]. In addition to these reports, results of other investigators demonstrated a modest association between C-Reactive Protein (CRP) and carotid artery stenosis and IMT [1,5,6]. As we know from previous studies, atherosclerosis considered to reflect chronic inflammation in arterial walls [7,8]. Patients cancidate for Coronary Artery Bypass Grafting (CABG) have higher level of atherosclerosis, so we aimed to determine the Common Carotid Intima-Media Thickness (CCIMT), High Sensitivity C-Reactive Protein (hsCRP) level, and their relationship in CABG candidates.

## Methods

### Study population

The study subjects were enrolled from consecutive patients of the Shahid Madani Hospital (Tabriz, Iran), who have been candidate for elective CABG from January 2005 to August 2007. In our referral center, all of CABG candidates underwent carotid and femoral arteries Doppler study, preoperatively.

Patients who had carotid endartrectomy, collagen-vascular diseases, diabetes, infection diseases history during recent month, inflammatory gastroenterology diseases, multiple sclerosis, liver diseases, end stage renal disease and previous stroke were excluded. The base-line examination included a medical history and laboratory testing.

### Ethical consideration

The study was performed according to the guidelines of Helsinki declarartion and approved by the Tabriz University of Medical Sciences Ethics Committee. All participants gave informed consent before including to the study.

### Carotid ultrasonographic evaluation

The common carotid arteries were evaluated with high-resolution B-mode ultrasonography using a 7.5- MHz linear-array transducer (Hitachi model EUB-525; Hitachi Medical Corp., Tokyo, Japan) to determine the IMT, site of plaques, and stenosis grade. Participants were examined in a supine position, with the head in the axis of the body slightly tilted to the either side. The IMT was measured in common carotid artery at sites 1.0 and 3.0 cm proximal from the beginning of carotid bulb and at the bottom of the bulb. The IMT was defined as the distance between two parallel echogenic lines corresponding to the blood-intima and media-adventitia interfaces. The average value of the 3 points was calculated for each side and the largest value (maximum IMT) was used for analysis.

The common carotid arteries were scanned cross-sectionally and longitudinally. All ultrasound measurements were recorded in end diastole and were measured by the investigator blinded to patients' clinical state. Plaque was defined as a regional intimal thickening, more than 1.2 mm in IMT height. In patients with plaque, carotid artery stenosis was graded using the peak systolic velocity measured after the plaque site according to the Society of Radiologists in Ultrasound Consensus Criteria [9].

### Serum hsCRP assay

At baseline examination, a blood sample from every participant was drawn and centrifuged at 1500 rpm for 5 minutes, and then the serum samples were stored at -70°C. All of samples were thawed together, and hsCRP was determined using commercially available enzyme linked immunosorbent assay kit (Monobind, Inc., Lake Forest, CA, USA; Lot Num: EIA-31K2A1).

### Statistical methods

Data analysis was performed using the SPSS package version 13 (Chicago, IL, USA). Continuous variables were compared between groups using parametric independent sample *t*-test, and oneway-ANOVA, or nonparametric Man-Whitney U test, and Kruskal walls. X^2 ^test also was used to analysis significant difference of categorized data. Pearson correlation and multiple regression were applied to evaluate the relationship of hsCRP level with CCIMT in patients without stenosis. A value of *p *< 0.05 was considered significant.

## Results

**Figure 1:**
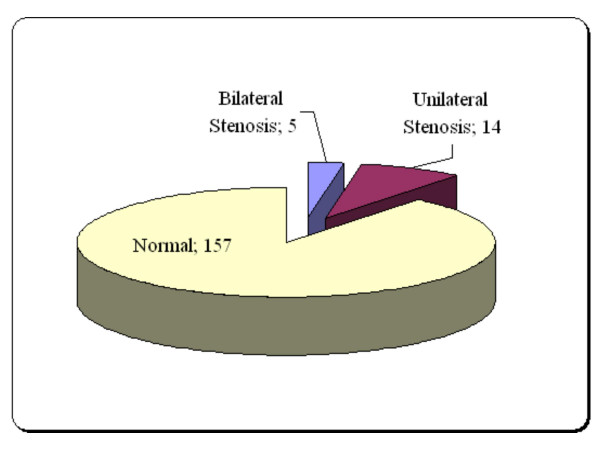
Frequency of common carotid artery stenosis in study population.

Finally, information of 176 CABG candidates was analysed. Demographic charcterstics of participants were listed in Table 1. Frequency of various grades of common carotid stenosis was shown in Table 2 based on right and left sides. Figure 1 demonstrated frequency of common carotid artery stenosis in study population. The mean of CCIMT was 0.69 ± 0.54 mm.

**Table 1:** Demographic characteristics of participants.

Variable		Value
Age (years)		62.71 ± 9.45
BMI (Kg/m^2^)		28.31 ± 3.74
Gender	Male	109 (61.93%)
	Female	67 (38.07%)
Male/female ratio		1.63
Smoking history	Male	58 (54.12%)
	Female	6(8.95%)
Smoking (Current/Former)	Male	13(11.92%)/45(42.19%)
	Female	0(0.0%)/6(8.95%)
Systolic blood pressure (mm Hg)		148.32 ± 24.43
Diastolic blood pressure (mm Hg)		91.54 ± 18.26
Anti-hypertensive therapy		70.45%
Lipid-lowering agent intake		46.02%

**Table 2:** Categorization of patients according to the stenosis grade of Common Carotid Artery (CCA).

	Grade of stenosis				
	
	Normal	< 50%	50–69%	70%-near to occlusion	Occlusion
Right CCA	166	7	2	1	-
Left CCA	162	10	2	2	-

Although there was no significant correlation between serum hsCRP level and CCIMT (*p *= 0.113, r = 0.186), participants with stenosis had higher levels of serum hsCRP than participants without stenosis in common carotid artery (2.42 ± 1.30 vs. 1.20 ± 0.97 mg/dl; *p *= 0.009).

In comparison of serum hsCRP levels among the various stenosis grades of common carotid artery, there were no significant difference between patients with < 50% stenosis, 50–60% stenosis, and 70-to near occlusion (*p *> 0.05; Table 3).

**Table 3:** hsCRP by stenosis grades.

	Stenosis grades of common carotid artery			
	
	Normal	< 50%	50–69%	70% to near occlusion
Serum hsCRP (mg/dl)	1.21 ± 0.97	2.18 ± 1.22	2.49 ± 1.25	2.27 ± 1.42

There was a direct linear correlation between age and CCIMT (*p *= 0.016, r = 0.239) in participants whitout stenosis.

## Discussion

This study examined the association of hsCRP with CCIMT. Study results showed that there was no correlation between hsCRP and CCIMT in CABG candidates with subclinical atherosclerosis. Despite this fact, patients with common carotid artery stenosis had higher levels of hsCRP than patients without stenosis. During present study, we strove to control conventional cardiovascular risk factors and diseases which may be affected hsCRP level. Furthermore, this study showed a lower incidence of severe carotid stenosis in Iranian population who candidate for CABG; like previous report of Tarzamni et al. for Iranian [10], and in contrast to the previous studies for other populations [11-14].

Several epidemiological studies have shown a link between serum hsCRP level and subsequent cardiovascular disease in general population [15-19]. In respect to the carotid atherosclerosis, an association between CRP levels and the presence of carotid plagues has been demonstrated by univariate analysis in a prospective studies conducted in general community, or in healthy middle aged women with a smoking history [20-22]. However, this relationship between IMT and CRP level appeared to be largely dependant on age or the presence of other cardiovascular risk factors. In contrast to the previous studies finding, other studies which carried out under controlled criteria failed to show a relation between CRP and carotid IMT and plaque formation [23,24].

To the best of our knowledge, this study is the first one that evaluated relationship of hsCRP and CCIMT among the CABG candidates. Study findings, in contrary with some previous reports [6,25], demonstrated that elevated level of hsCRP was not associated with increased IMT. While patients with carotid artery stenosis had higher level of serum hsCRP, there was not statistcally significant difference between various grades of stenosis. As we know, patients who candidate for CABG have higher grades of atherosclerosis in coronary arteries. Today, atherosclerosis consider as a chronic inflammatory process [7,8], and innate and adaptive immune responses participate in several phases of atherosclerosis [26]. CRP is a component of innate immunity that is actively participates in inflammatory process. Recent evidences have suggested a possible direct pathogenic role for CRP in atherosclerosis process and plaque formation [27-29] and increasing of CRP level promote arterial atherosclerosis. Alternatively, it may merely be an epiphenomena and an indicator of systemic inflammation which itself is associated with atherosclerosis.

Furthermore, some of conventional cardiovascular risk factors such as smoking, obesity may act to increase atherosclerosis partly through increasing systemic inflammation. The systemic inflammatory response is also increased in smokers according to the pack pear year of smoking [23]. This may be related to both of bacterial endotoxin and the increased risk of respiratory tract infection seen in smokers [30]. Therefore, elevated CRP may merely reflect an exaggerated inflammatory response associated with these conventional cardiovascular risk factors [31,32].

As well-known ligand of phosphorylcholine residues, CRP binds avidly to oxidized low-density lipoproteins and may induce foam cell formation in atherosclerosis [33,34]. In this case and by considering the patients with carotid artery stenosis had higher serum hsCRP levels, the CRP effect might be confirmed clinically by study results.

Despite these findings, one of our study limitations was limited population. So, we struggled to use proper statistical tests to analysis data and appropriate inclusion and exclusion criteria to control any factors may address any bias.

## Conclusion

In conclusion, our study results showed that there was no correlation between serum hsCRP level and intima-media thickness of common carotid in patients without stenosis, but patients with common carotid artery stenosis had higher levels of serum hsCRP than patients without stenosis.

## Abbreviations

IMT: Intima-Media Thickness; CRP: C-Reactive Protein, hsCRP: High-Sensitivity C-Reactive Protein; CABG: Coronary Artery Bypass Grafting; CCIMT: Common Carotid Intima-Media Thickness

## Competing interests

The authors declare that they have no competing interests.

## Authors' contributions

PS designed study and performed Doppler ultrasonographic evaluation.

NN followed up the patients, performed data analysis, drafted and revised the manuscript

MKT conceived the paper, and carried out the Doppler ultrasound

RJR participated in coordination and enrolment of patients to the study

All authors read and approved the final manuscript.
